# The Advisory Committee on Immunization Practices’ Interim Recommendation for Use of Pfizer-BioNTech COVID-19 Vaccine in Adolescents Aged 12–15 Years — United States, May 2021

**DOI:** 10.15585/mmwr.mm7020e1

**Published:** 2021-05-21

**Authors:** Megan Wallace, Kate R. Woodworth, Julia W. Gargano, Heather M. Scobie, Amy E. Blain, Danielle Moulia, Mary Chamberland, Nicole Reisman, Stephen C. Hadler, Jessica R. MacNeil, Doug Campos-Outcalt, Rebecca L. Morgan, Matthew F. Daley, José R. Romero, H. Keipp Talbot, Grace M. Lee, Beth P. Bell, Sara E. Oliver

**Affiliations:** ^1^CDC COVID-19 Response Team; ^2^Epidemic Intelligence Service, CDC; ^3^University of Arizona, College of Medicine, Phoenix, Arizona; ^4^Department of Health Research Methods, Evidence and Impact, Hamilton, Ontario; ^5^Institute for Health Research, Kaiser Permanente Colorado, Denver, Colorado; ^6^Arkansas Department of Health; ^7^Vanderbilt University School of Medicine, Nashville, Tennessee; ^8^Stanford University School of Medicine, Stanford, California; ^9^University of Washington, Seattle, Washington.

The Pfizer-BioNTech COVID-19 (BNT162b2) vaccine is a lipid nanoparticle–formulated, nucleoside-modified mRNA vaccine encoding the prefusion spike glycoprotein of SARS-CoV-2, the virus that causes COVID-19. Vaccination with the Pfizer-BioNTech COVID-19 vaccine consists of 2 intramuscular doses (30 *μ*g, 0.3 mL each) administered 3 weeks apart. On December 11, 2020, the Food and Drug Administration (FDA) issued an Emergency Use Authorization (EUA) for use of the Pfizer-BioNTech COVID-19 vaccine (Pfizer, Inc; Philadelphia, Pennsylvania) in persons aged ≥16 years (*1*); on December 12, 2020, the Advisory Committee on Immunization Practices (ACIP) issued an interim recommendation for use of the vaccine in the same age group (*2*). As of May 12, 2021, approximately 141.6 million doses of the Pfizer-BioNTech COVID-19 vaccine had been administered to persons aged ≥16 years.* On May 10, 2021, FDA expanded the EUA for the Pfizer-BioNTech COVID-19 vaccine to include adolescents aged 12–15 years (*1*). On May 12, 2021, ACIP issued an interim recommendation^†^ for use of the Pfizer-BioNTech COVID-19 vaccine in adolescents aged 12–15 years for the prevention of COVID-19. To guide its deliberations regarding the vaccine, ACIP used the Evidence to Recommendation (EtR) Framework,^§^ using the Grading of Recommendations, Assessment, Development and Evaluation (GRADE) approach.^¶^ The ACIP recommendation for the use of the Pfizer-BioNTech COVID-19 vaccine in persons aged ≥12 years under an EUA is interim and will be updated as additional information becomes available.

Since June 2020, ACIP has convened 14 public meetings to review data on the epidemiology of COVID-19 and the potential use of COVID-19 vaccines, including the Pfizer-BioNTech COVID-19 vaccine (*3*). The ACIP COVID-19 Vaccines Work Group, comprising experts in infectious diseases, vaccinology, vaccine safety, public health, and ethics, has held weekly meetings to review COVID-19 surveillance data, evidence for vaccine efficacy and safety, and implementation considerations for COVID-19 vaccines. Within the EtR Framework for the Pfizer-BioNTech COVID-19 vaccine for adolescents aged 12–15 years, ACIP considered the importance of COVID-19 as a public health problem, as well as issues of resource use, benefits and harms, patients’ and parents’ values and preferences, acceptability, feasibility, and equity for use of the vaccine among adolescents. After a systematic review of published and unpublished evidence for benefits and harms, the Work Group used the GRADE approach to assess the certainty of evidence for outcomes related to the vaccine, rated on a scale of 1 (high certainty) to 4 (very low certainty) (*4*). Work Group conclusions regarding the evidence for the Pfizer-BioNTech COVID-19 vaccine were presented to ACIP at a public meeting on May 12, 2021.

The body of evidence for the Pfizer-BioNTech COVID-19 vaccine was primarily guided by one randomized, double-blind, placebo-controlled Phase II/III clinical trial that was expanded to enroll approximately 2,200 participants aged 12–15 years, randomized 1:1 to receive vaccine or saline placebo (*5*). Interim findings from this clinical trial were based on data from participants with a median of 2 months of follow-up. The estimated efficacy of the Pfizer-BioNTech COVID-19 vaccine was supported by two types of evidence: clinical efficacy and immunobridging. In the direct clinical assessment, efficacy was 100% (95% confidence interval [CI] = 75.3%–100%) in preventing symptomatic, laboratory-confirmed COVID-19 in adolescents aged 12–15 years without evidence of previous SARS-CoV-2 infection. Vaccine efficacy was also supported by immunobridging data from vaccine recipients aged 12–15 years compared with those from recipients aged 16–25 years. The immune response to 2 doses of the Pfizer-BioNTech COVID-19 vaccine in adolescents aged 12–15 years without evidence of previous SARS-CoV-2 infection was at least as high as the response observed in persons aged 16–25 years; the geometric mean ratio for 50% neutralizing antibody titer was 1.76 (95% CI = 1.47–2.10), demonstrating statistical noninferiority.** Among adolescent vaccine recipients aged 12–15 years, reactogenicity symptoms, defined as solicited local injection site or systemic reactions during the 7 days after vaccination, were frequent (90.9% of vaccine recipients reported any local reaction, and 90.7% reported any systemic reaction) and mostly mild to moderate. Systemic adverse reactions were more commonly reported after the second dose than after the first dose, had a median onset of 1–4 days after vaccine receipt, and resolved in a median of 1–2 days. Severe local and systemic adverse reactions (grade ≥3, defined as interfering with daily activity) occurred more commonly in vaccine recipients than in placebo recipients. Among vaccine recipients, 10.7% reported any reaction of grade ≥3; the most common symptoms were fatigue (3.5%), fever (3.0%), headache (2.7%), chills (2.1%), and injection-site pain (1.5%). Overall, reactions of grade ≥3 were also more commonly reported after the second dose than after the first dose. The frequency of serious adverse events^††^ was low among all participants; five serious adverse events (0.4%) were reported among vaccine recipients and two (0.2%) among placebo recipients, with no statistically significant difference in frequency observed between the two groups (*5*). These serious adverse events encompassed medical events occurring at a frequency similar to that in the general population aged 12–15 years, with none considered to be related to vaccination (*5*). No specific safety concerns were identified among adolescent vaccine recipients. A detailed summary of safety data, including information on reactogenicity, is available at https://www.cdc.gov/vaccines/covid-19/info-by-product/pfizer/reactogenicity.html.

From the GRADE evidence assessment, the level of certainty for the benefits of Pfizer-BioNTech COVID-19 vaccination among adolescents aged 12–15 years was type 1 (high certainty) for the prevention of symptomatic COVID-19. Regarding potential harms after vaccination, evidence was type 4 (very low certainty) for serious adverse events and type 1 (high certainty) for reactogenicity. No data were available to assess the other GRADE benefits and harms including prevention of hospitalization due to COVID-19, prevention of multisystem inflammatory syndrome in children (MIS-C), SARS-CoV-2 seroconversion to a nonspike protein, or prevention of asymptomatic SARS-CoV-2 infection.

Data reviewed within the EtR Framework supported the use of the Pfizer-BioNTech COVID-19 vaccine in adolescents aged 12–15 years. ACIP determined that COVID-19 in adolescents is a major public health problem. Adolescents represent a growing proportion of new COVID-19 cases reported to CDC^§§^ and have been shown to contribute to household transmission (*6*). As of May 1, 2021, the cumulative COVID-19–associated hospitalization rate for adolescents aged 12–17 years was 51.3 per 100,000 population,^¶¶^ which is higher than the influenza-associated hospitalization rate for the same age group during the 2009 H1N1 influenza pandemic (23.9 per 100,000 population).*** As of May 3, 2021, CDC had received reports of 3,742 cases of MIS-C, a severe hyperinflammatory syndrome occurring several weeks after acute SARS-CoV-2 infection; 21.5% of the MIS-C cases have occurred in adolescents aged 12–17 years.^†††^ ACIP determined that use of the Pfizer-BioNTech COVID-19 vaccine among adolescents is a reasonable and efficient allocation of resources. Whereas there might be uncertainty regarding how different populations value the vaccine, results from several surveys suggest that approximately one half of parents were willing to have their adolescent children vaccinated (range = 46%–60%).^§§§^ Overall, ACIP determined that the desirable effects clearly outweighed any undesirable effects in most settings. In expanding COVID-19 vaccine access, additional considerations should be given to demographic groups with disproportionate COVID-19 morbidity and mortality, as well as those with barriers to routine health care (e.g., adolescents of certain racial/ethnic groups and those living in a rural or frontier area, experiencing homelessness, having a disability, or lacking health insurance). Providing rapid and equitable access to COVID-19 vaccine for adolescents will require a stepwise approach, including augmenting existing infrastructure for vaccination, increasing enrollment of providers caring for adolescents into the COVID-19 vaccination program, and applying school-focused strategies to ensure vaccination opportunities for a diverse population. Some aspects of the Pfizer-BioNTech COVID-19 vaccine (e.g., cold-chain storage requirements or large minimum order sizes) might limit access to the vaccine among some populations, which could negatively affect health equity. Advancing health equity, particularly in populations that experience disproportionate COVID-19 morbidity and mortality, requires engagement with community leaders, adolescent health care providers, and parents to identify and remove barriers to COVID-19 vaccination, including those related to vaccine access and vaccine confidence. The GRADE evidence profile and EtR supporting evidence are available at https://www.cdc.gov/vaccines/acip/recs/grade/covid-19-pfizer-biontech-vaccine-12-15-years.html and https://www.cdc.gov/vaccines/acip/recs/grade/covid-19-pfizer-biontech-etr-12-15-years.html.

Before vaccination, the EUA Fact Sheet should be provided to recipients and parents or guardians. There is no federal, legal requirement for caregiver consent for COVID-19 vaccination or any other vaccination; however, COVID-19 vaccine must be administered according to applicable state and territorial vaccination laws. Providers should counsel Pfizer-BioNTech COVID-19 vaccine recipients and parents or guardians about expected systemic and local reactogenicity. Additional clinical considerations are available at https://www.cdc.gov/vaccines/covid-19/info-by-manufacturer/pfizer/clinical-considerations.html. The interim recommendation and clinical considerations are based on use of the Pfizer-BioNTech COVID-19 vaccine under an EUA and might change as more evidence becomes available. ACIP will continue to review additional data as they become available; updates to recommendations or clinical considerations will be posted on the ACIP website (https://www.cdc.gov/vaccines/hcp/acip-recs/vacc-specific/covid-19.html).

## Reporting of Vaccine Adverse Events

FDA requires that vaccination providers report vaccination administration errors, serious adverse events, cases of multisystem inflammatory syndrome, and cases of COVID-19 that result in hospitalization or death after administration of COVID-19 vaccine under an EUA (*7*). Adverse events that occur after receipt of any COVID-19 vaccine should be reported to the Vaccine Adverse Events Reporting System (VAERS). Information on how to submit a report to VAERS is available at https://vaers.hhs.gov/index.html or 1-800-822-7967. Any person who administers or receives a COVID-19 vaccine is encouraged to report any clinically significant adverse event, whether or not it is clear that a vaccine caused the adverse event. In addition, CDC has developed a new, voluntary smartphone-based online tool (v-safe) that uses text messaging and online surveys to provide near real-time health check-ins after receipt of a COVID-19 vaccine. Parents or guardians can register their adolescent children in v-safe and complete the health surveys on their behalf. CDC’s v-safe call center follows up on reports to v-safe that include possible medically significant health events to collect additional information for completion of a VAERS report. Information on v-safe is available at https://www.cdc.gov/vsafe.

**Summary**

**What is already known about this topic?**

On May 10, 2021, the Food and Drug Administration expanded Emergency Use Authorization for the Pfizer-BioNTech COVID-19 vaccine to include adolescents aged 12–15 years.

**What is added by this report?**

On May 12, 2021, after a systematic review of all available data, the Advisory Committee on Immunization Practices made an interim recommendation for use of the Pfizer-BioNTech COVID-19 vaccine in adolescents aged 12–15 years for the prevention of COVID-19.

**What are the implications for public health practice?**

The Pfizer-BioNTech COVID-19 vaccine is the first COVID-19 vaccine approved for use in adolescents and has high efficacy against symptomatic COVID-19. Vaccination will be important to protect adolescents against symptomatic COVID-19 disease and to reduce community transmission of SARS-CoV-2.

## Footnotes

* Accessed May 12, 2021. https://covid.cdc.gov/covid-data-tracker/#vaccinations

† On May 12, 2021, ACIP voted 14–0 (one recusal) in favor of the interim recommendation for use of Pfizer BioNTech COVID-19 vaccine for persons aged 12–15 years. One ACIP member recused herself because of participation in clinical trials and other studies involving companies producing COVID-19 vaccines.

§
https://www.cdc.gov/vaccines/acip/recs/grade/downloads/ACIP-evidence-rec-frame-508.pdf

¶
https://www.cdc.gov/vaccines/acip/recs/grade/about-grade.html

** 1.5-fold noninferiority criterion: lower bound of the two-sided 95% CI for geometric mean ratio >0.67.

†† Serious adverse events are defined as any untoward medical occurrence that results in death, is life-threatening, requires inpatient hospitalization or prolongation of existing hospitalization, or results in persistent disability/incapacity.

§§
https://covid.cdc.gov/covid-data-tracker/#demographicsovertime

¶¶
https://gis.cdc.gov/grasp/COVIDNet/COVID19_3.html

***
https://gis.cdc.gov/GRASP/Fluview/FluHospRates.html

†††
https://www.cdc.gov/mis-c/cases/index.html

§§§
https://www.cdc.gov/vaccines/acip/recs/grade/covid-19-pfizer-biontech-etr-12-15-years.html
